# Health-related quality of life of postmenopausal women with hormone receptor-positive, human epidermal growth factor receptor 2-negative advanced breast cancer treated with ribociclib + letrozole: results from MONALEESA-2

**DOI:** 10.1007/s10549-018-4769-z

**Published:** 2018-04-13

**Authors:** Sunil Verma, Joyce O’Shaughnessy, Howard A. Burris, Mario Campone, Emilio Alba, David Chandiwana, Anand A. Dalal, Santosh Sutradhar, Mauricio Monaco, Wolfgang Janni

**Affiliations:** 1 Department of Oncology, Cumming School of Medicine, Tom Baker Cancer Centre, University of Calgary, 1331 29th Street NW, Calgary, AB T2N 4N2 Canada; 2 Texas Oncology-Baylor Charles A. Sammons Cancer Center, Baylor University Medical Center, and The US Oncology Network, 3410 Worth Street, Suite 400, Dallas, TX 75246 USA; 3 Drug Development, Sarah Cannon Research Institute, 250 25th Avenue North, Suite 100, Nashville, TN 37203 USA; 4 Medical Oncology, Institut de Cancérologie de l’Ouest/René Gauducheau Centre de Recherche en Cancérologie, Boulevard Jacques Monod, Nantes, 44805 Saint‐Herblain France; 5 Medical Oncology Unit, Hospital Universitario Virgen de la Victoria, IBIMA, 29010 Málaga, Spain; 6 Novartis Pharmaceuticals Corporation, One Health Plaza, East Hanover, NJ 07936-1080 USA; 7 Department of Gynecology, Universitätsklinikum Ulm, Prittwitzstraße 43, 89075 Ulm, Germany

**Keywords:** CDK4/6 inhibitor, Ribociclib, Advanced breast cancer, Health-related quality of life, Endocrine therapy, Hormone receptor-positive

## Abstract

**Purpose:**

Evaluate patient-reported outcomes (PROs) for postmenopausal women with hormone receptor-positive (HR+), human epidermal growth factor receptor 2-negative (HER2−) advanced breast cancer treated with first-line ribociclib plus letrozole.

**Methods:**

In the phase III MONALEESA-2 study (NCT01958021), 668 patients were randomized 1:1 to ribociclib (600 mg/day; 3-weeks-on/1-week-off) plus letrozole (2.5 mg/day) or placebo plus letrozole. PROs were assessed using the European Organisation for Research and Treatment of Cancer core quality-of-life (EORTC QLQ-C30) and breast cancer-specific (EORTC QLQ-BR23) questionnaires. Changes from baseline and time to deterioration in health-related quality of life (HRQoL) were analyzed using linear mixed-effect and stratified Cox regression models, respectively. Exploratory analysis of area-under-the-curve for change from baseline in pain score (AUC-pain) was performed.

**Results:**

On-treatment HRQoL scores were consistently maintained from baseline and were similar between arms. A clinically meaningful (> 5 points) reduction in pain score was observed as early as Week 8 and was maintained up to Cycle 15 in the ribociclib arm. A statistically significant increase in mean AUC-pain was also observed in the ribociclib arm. Scores for all other EORTC QLQ-C30 and EORTC QLQ-BR23 domains were maintained from baseline and were similar between arms.

**Conclusions:**

HRQoL was consistently maintained from baseline in postmenopausal women with HR+, HER2− advanced breast cancer receiving ribociclib plus letrozole and was similar to that observed in the placebo plus letrozole arm. Together with the improved clinical efficacy and manageable safety profile, these PRO results provide additional support for the benefit of ribociclib plus letrozole in this patient population.

## Introduction

Ribociclib is an orally bioavailable, highly selective inhibitor of cyclin-dependent kinases (CDK) 4 and 6 (CDK4/6) [1]. In clinical studies, ribociclib has demonstrated significant activity together with endocrine therapy as a first-line treatment in hormone receptor-positive (HR+), human epidermal growth factor receptor 2-negative (HER2−) advanced breast cancer [2–4]. In the phase III MONALEESA-2 study, first-line treatment with ribociclib plus letrozole significantly prolonged progression-free survival (PFS) at the pre-planned interim analysis (hazard ratio: 0.556; 95% confidence interval [CI] 0.429–0.720; *p* = 0.00000329), showing higher overall response rates versus placebo plus letrozole in postmenopausal women with HR+, HER2− recurrent/metastatic breast cancer [4, 5]. An updated analysis demonstrated maintained treatment benefit with ribociclib plus letrozole: median PFS was 25.3 months versus 16.0 months in the placebo plus letrozole arm (hazard ratio: 0.568; 95% CI 0.457‒0.704; *p *= 0.0000000963) [6].

Targeted combination therapies are associated with higher response rates and delayed progression in patients with HR+, HER2− advanced breast cancer when compared with single-agent endocrine therapy, but this approach can expose patients to additional treatment-related toxicities, which can affect their quality of life (QoL) [7]. In general, CDK4/6 inhibitor-based regimens are associated with predictable and manageable safety profiles, with myelosuppression observed most commonly [4, 6, 8]. Recent guidelines recommend that the impact of treatment on QoL should be considered in addition to efficacy and safety [9]. In this analysis of the MONALEESA-2 study, we report validated patient-reported outcomes (PROs) results, including health-related QoL (HRQoL) and improvement in symptoms.

## Methods

### Study design and treatment

A detailed study design has previously been reported [5]. MONALEESA-2 is an ongoing, double-blind, randomized phase III study of first-line ribociclib (600 mg/day on a 3-weeks-on/1-week-off schedule) or placebo in combination with letrozole (2.5 mg/day on a continuous schedule). PROs were a secondary objective. The trial protocol was reviewed and approved by an independent ethics committee and institutional review board at each site. A steering committee oversaw the conduct of the study in conformation with the approved protocol. The study was conducted in accordance with the International Conference on Harmonisation’s Harmonised Tripartite Guideline for Good Clinical Practice, all applicable local regulations, and the ethical principles of the Declaration of Helsinki.

### PRO assessments

PRO measures of HRQoL, functioning, disease symptoms, and treatment-related side effects were assessed using the European Organisation for Research and Treatment of Cancer core quality-of-life (EORTC QLQ-C30; v3.0) [10] and breast cancer-specific (EORTC QLQ-BR23; v1.0) questionnaires [11].

Patients were asked to complete both questionnaires at the beginning of each visit at screening, every 8 weeks for the first 18 months, then every 12 weeks thereafter until disease progression, death, loss to follow-up, or withdrawal of consent, and at treatment discontinuation. Questionnaire responses were converted to a score ranging from 0 to 100. For functional and global health status/QoL scales, a higher numerical score represents a better level of functioning/HRQoL; a positive change from baseline was considered an improvement in functioning/HRQoL. For symptomatic scales, a higher numerical score represents greater symptom severity; a negative change from baseline was considered an improvement in symptom severity.

### Statistical analyses

PRO analyses were based on the full analysis set (*N* = 668), following the intent-to-treat principle. For partially completed multi-item scales, scores were equal to the average of the completed items for that particular respondent. Changes from baseline were analyzed using a linear mixed-effect model. Evaluable patients had baseline scores and at least one non-missing postbaseline PRO assessment. A post hoc analysis of time to definitive deterioration (TTD) in overall HRQoL EORTC QLQ-C30 scores by ≥ 10% was performed for patients with, versus without, a PFS event, within each treatment arm, and among all treated patients across both arms using the stratified log-rank test with a two-sided *p*-value. A definitive deterioration event was defined as a decrease of ≥ 10% in EORTC QLQ-C30 global health status/QoL score relative to baseline, with no subsequent improvement above this threshold, or death due to any cause. Patients with no definitive deterioration events were censored at the date of the last available PRO assessment. HRQoL deterioration was considered clinically meaningful using previously established thresholds for minimally important differences (MID) in QoL; for EORTC QLQ-C30, the threshold for MID was a change of 5–10 points from baseline [12]. The Kaplan–Meier method was used to estimate the median TTD in HRQoL by ≥ 10%; hazard ratio and two-sided 95% CIs were estimated using a stratified Cox regression model. Exploratory analysis of area-under-the-curve (AUC) for change from baseline in EORTC QLQ-C30 pain scores (AUC-pain) was also performed; mean AUC-pain was compared between the two treatment arms using a t-test. No multiplicity adjustments were made for *p*-values for exploratory and subgroup analyses.

## Results

### Patient characteristics and disposition

A total of 668 patients were randomized to ribociclib (600 mg/day on a 3-weeks-on/1-week-off schedule) plus letrozole (2.5 mg/day on a continuous schedule; *n *= 334) or placebo plus letrozole (*n* = 334) [4]. Baseline patient and disease characteristics were well balanced across treatment arms (Table 1).

**Table 1:** Patient demographics and baseline characteristics [4]

Patient/baseline characteristic	Ribociclib + letrozole *n* = 334	Placebo + letrozole *n* = 334
Median age, years (range)	62 (23–91)	63 (29–88)
Race, *n* (%)		
White	269 (80.5)	280 (83.8)
Asian	28 (8.4)	23 (6.9)
Black	10 (3.0)	7 (2.1)
Other/unknown	27 (8.1)	24 (7.2)
ECOG PS, *n* (%)		
0	205 (61.4)	202 (60.5)
1	129 (38.6)	132 (39.5)
Metastatic sites, *n* (%)		
Visceral disease	197 (59.0)	196 (58.7)
Bone-only disease	69 (20.7)	78 (23.4)
De novo metastatic disease, *n* (%)	114 (34.1)	113 (33.8)
Prior (neo)adjuvant therapy, *n* (%)^a^		
Chemotherapy	146 (43.7)	145 (43.4)
Endocrine therapy^b^	175 (52.4)	171 (51.2)

Data cut-off: January 29, 2016

*ECOG PS* Eastern Cooperative Oncology Group performance status

^a^Some patients received both chemotherapy and endocrine therapy as neoadjuvant or adjuvant treatment

^b^Endocrine therapy includes anastrozole, exemestane, goserelin, letrozole, tamoxifen, and treatments coded as “other”

Measurable disease data were based on a cut-off date of January 2, 2017. Patient demographics, disposition, and EORTC questionnaire completion data were based on a cut-off date of January 29, 2016. All PRO data were based on a cut-off date of January 4, 2017.

### EORTC QLQ-C30 global health status/QoL scale

Overall compliance rates of patients completing the HRQoL questionnaires during the treatment period were high in both treatment arms (Table 2).

**Table 2:** EORTC QLQ-C30 and EORTC QLQ-BR23 questionnaire completion rates

	Ribociclib + letrozole *n* = 334		Placebo + letrozole *n *= 334	
	Patients on study at scheduled day, *n*^a^	Patients on study at scheduled day with valid questionnaire within time window, *n* (%)^b^	Patients on study at scheduled day, *n*^a^	Patients on study at scheduled day with valid questionnaire within time window, *n* (%)^b^
EORTC QLQ-C30 completion rates				
Baseline	334	324 (97.0)	334	327 (97.9)
Cycle 3 Day 1	309	293 (94.8)	298	291 (97.7)
Cycle 5 Day 1	283	269 (95.1)	273	264 (96.7)
Cycle 7 Day 1	268	257 (95.9)	259	255 (98.5)
Cycle 9 Day 1	248	237 (95.6)	236	227 (96.2)
Cycle 11 Day 1	236	230 (97.5)	215	202 (94.0)
Cycle 13 Day 1	216	206 (95.4)	195	186 (95.4)
Cycle 15 Day 1	171	154 (90.1)	136	133 (97.8)
Cycle 17 Day 1	118	110 (93.2)	89	81 (91.0)
Cycle 19 Day 1	69	61 (88.4)	45	41 (91.1)
Cycle 22 Day 1	20	17 (85.0)	17	13 (76.5)
Cycle 25 Day 1	4	3 (75.0)	–	–
EORTC QLQ-BR23 completion rates				
Baseline	334	324 (97.0)	334	326 (97.6)
Cycle 3 Day 1	308	294 (95.5)	298	289 (97.0)
Cycle 5 Day 1	283	269 (95.1)	273	265 (97.1)
Cycle 7 Day 1	268	257 (95.9)	259	254 (98.1)
Cycle 9 Day 1	248	237 (95.6)	236	228 (96.6)
Cycle 11 Day 1	236	230 (97.5)	215	203 (94.4)
Cycle 13 Day 1	216	206 (95.4)	195	184 (94.4)
Cycle 15 Day 1	171	153 (89.5)	136	131 (96.3)
Cycle 17 Day 1	118	110 (93.2)	89	80 (89.9)
Cycle 19 Day 1	69	61 (88.4)	45	42 (93.3)
Cycle 22 Day 1	20	17 (85.0)	17	13 (76.5)
Cycle 25 Day 1	4	3 (75.0)	–	–

*EORTC QLQ*-*BR23* European Organisation for Research and Treatment of Cancer breast cancer-specific questionnaire, *EORTC QLQ*-*C30* European Organisation for Research and Treatment of Cancer core quality-of-life questionnaire, *QoL* quality of life

^a^Number of patients eligible to complete the questionnaire at the corresponding visit

^b^At least one valid score among QoL, physical functioning, emotional functioning, and social functioning scores was required for the EORTC QLQ-C30 questionnaire

**Fig. 1:**
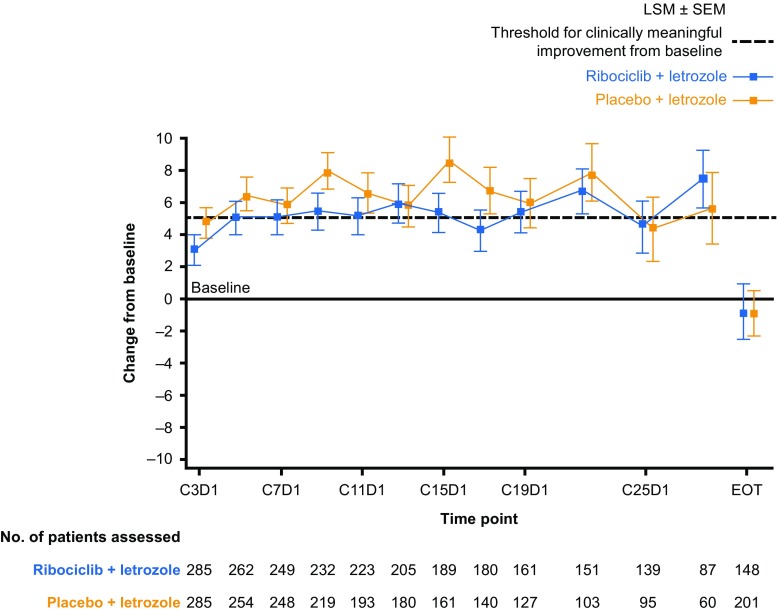
Overall change from baseline in patient-reported EORTC QLQ-C30 global health status/QoL scores by treatment. *C* Cycle, *D* Day, *EORTC QLQ*-*C30* European Organisation for Research and Treatment of Cancer core quality-of-life questionnaire, *EOT* end of treatment, *HRQoL* health-related quality of life, *LSM* least squares mean, *QoL* quality of life, *SEM* standard error of the mean. Changes from baseline in patient-reported EORTC QLQ-C30 global health status/QoL scores were determined using a linear mixed-effect model. Positive changes from baseline indicate improvement in HRQoL. A > 5-point improvement from baseline in HRQoL score was defined as clinically meaningful. Data cut-off: January 4, 2017

**Fig. 2:**
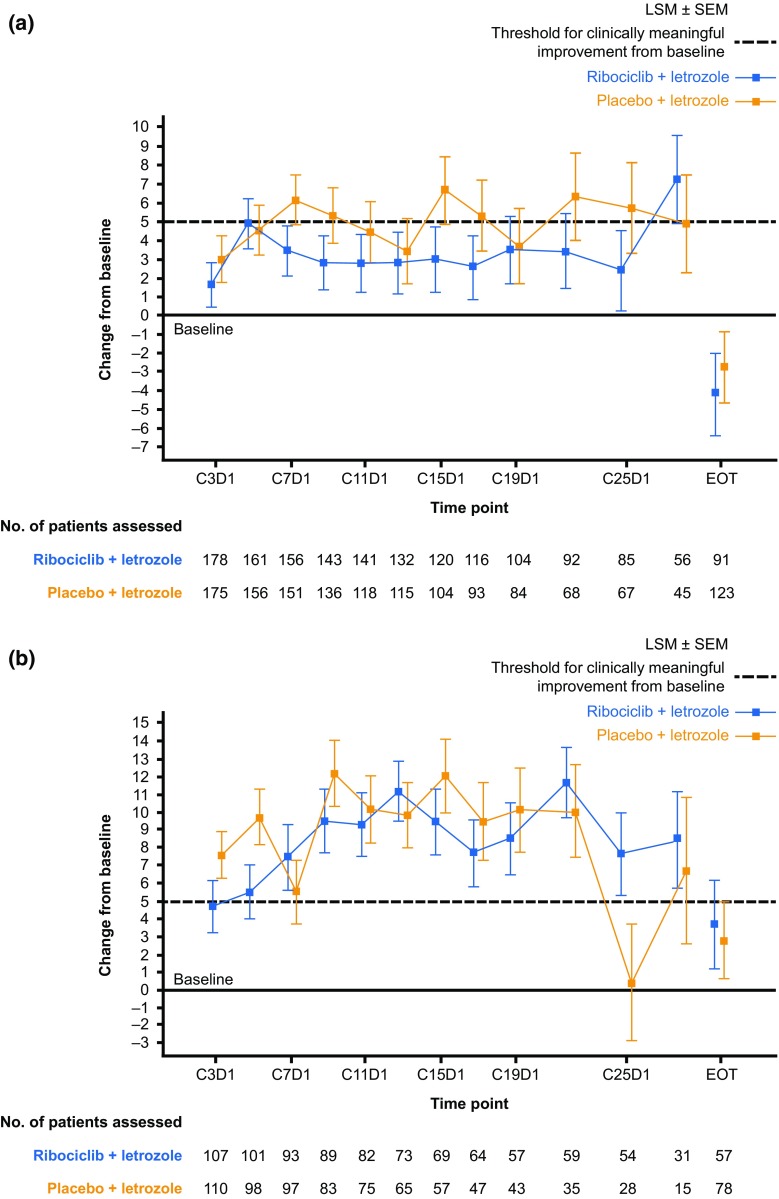
Overall change from baseline in patient-reported EORTC QLQ-C30 global health status/QoL scores in patients with a baseline ECOG PS of 0 (**a**) or 1 (**b**). *C* Cycle, *D* Day, *ECOG PS* Eastern Cooperative Oncology Group performance status, *EORTC QLQ*-*C30* European Organisation for Research and Treatment of Cancer core quality-of-life cancer questionnaire, *EOT* end of treatment, *HRQoL* health-related quality of life, *LSM* least square mean, *QoL* quality of life, *SEM* standard error of the mean. Changes from baseline in patient-reported EORTC QLQ-C30 global health status/QoL scores were determined using a linear mixed-effect model. Positive changes from baseline indicate improvement in HRQoL. A > 5-point improvement from baseline in HRQoL score was defined as clinically meaningful. Data cut-off: January 4, 2017

EORTC QLQ-C30 global health status/QoL scores were consistently maintained from baseline and were similar in both treatment arms during the study treatment period, with clinically meaningful (> 5 points from baseline) improvements observed at some timepoints (Fig. 1). Differences between treatment arms in overall HRQoL were less than the MID. TTD by ≥ 10% in overall HRQoL was also similar between treatment arms (hazard ratio: 0.944; 95% CI 0.720–1.237) [13]. Mean overall HRQoL worsened in both treatment arms at end of treatment (EOT) despite the earlier improvements from baseline (Fig. 1). Overall HRQoL was also maintained from baseline in both treatment arms during the study treatment period in subgroups of patients with bone-only metastases, visceral disease, those with a best overall response of complete response (CR) or partial response (PR; data not shown), and those with an Eastern Cooperative Oncology Group performance status of 0 or 1 (Fig. 2).

**Fig. 3:**
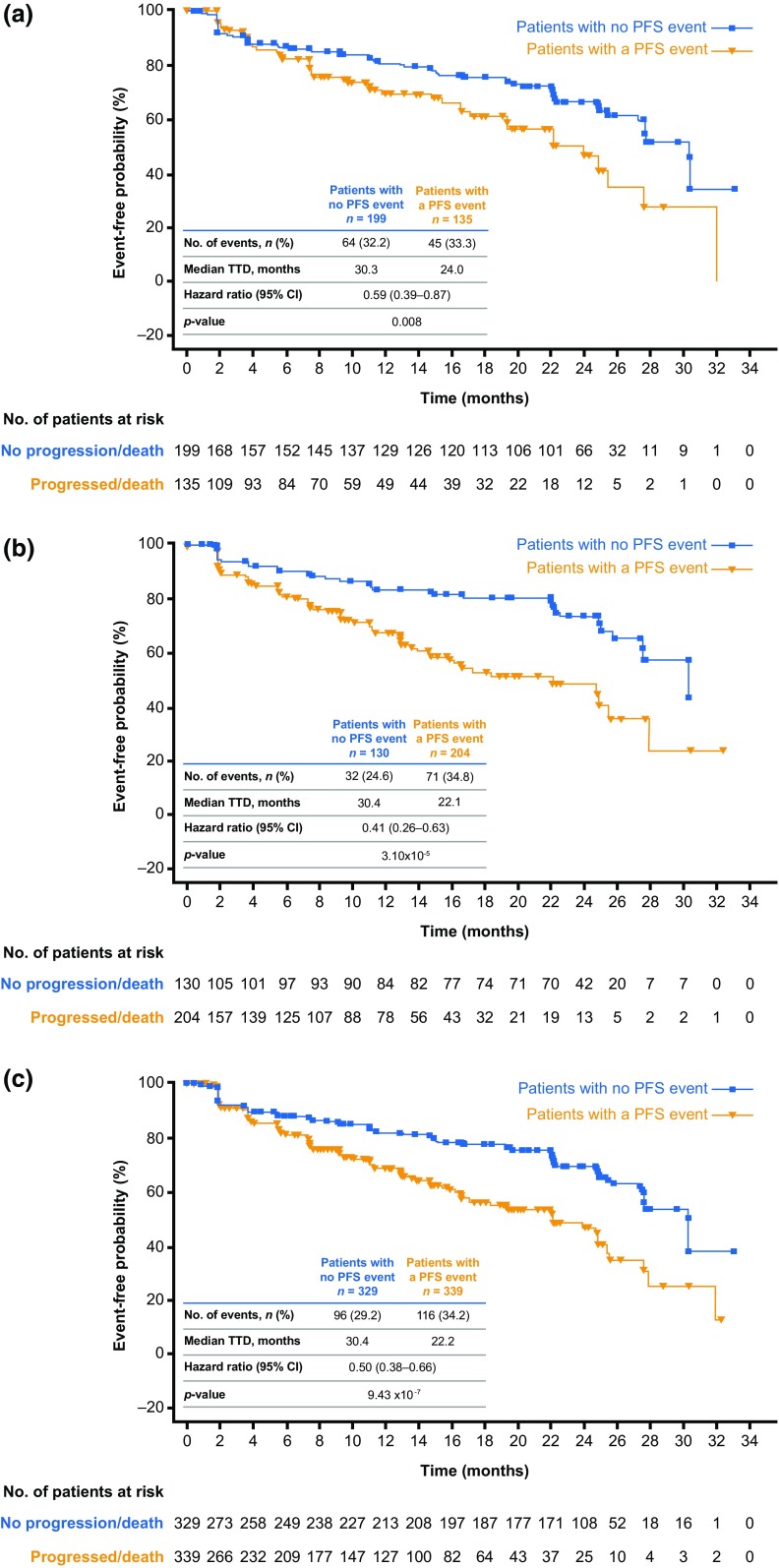
Time to definitive deterioration of global health status/QoL scale score of EORTC QLQ-C30 from baseline by ≥ 10% in patients with or without a PFS event (disease progression or death) in the ribociclib plus letrozole arm (**a**), placebo plus letrozole arm (**b**), and in all treated patients across both arms (**c**). *CI* confidence interval, *EORTC QLQ*-*C30* European Organisation for Research and Treatment of Cancer core quality-of-life questionnaire, *PFS* progression-free survival, *QoL* quality of life, *TTD* time to definitive deterioration. Data cut-off: January 4, 2017

According to post hoc analyses, a significantly greater delay in TTD (≥ 10% decrease) in overall HRQoL was observed in patients who did not experience a PFS event versus those who did experience a PFS event (disease progression or death; Fig. 3). Delay in HRQoL deterioration was observed in both the ribociclib plus letrozole arm (hazard ratio: 0.59; 95% CI 0.39–0.87; *p *= 0.008; Fig. 3a) and placebo plus letrozole arm (hazard ratio: 0.41; 95% CI 0.26–0.63; *p *= 0.000031; Fig. 3b). The delay in HRQoL deterioration in patients without, versus with, a PFS event was similar in the population of all treated patients (hazard ratio: 0.50; 95% CI 0.38–0.66; *p *= 0.000000943; Fig. 3c).

### EORTC QLQ-C30 symptom scales

Mean baseline scores for EORTC QLQ-C30 symptoms, including fatigue [30.9 (standard deviation [SD]: 23.9) versus 31.4 (SD: 24.2) in the ribociclib versus placebo arms, respectively], nausea and vomiting [7.3 (SD: 15.3) versus 8.6 (SD: 17.8)], and diarrhea [8.1 (SD: 16.8) versus 7.1 (SD: 16.4)] were generally at the lower end of the score range in both treatment arms, indicating lower symptom severity.

During study treatment, HRQoL was maintained in patients experiencing fatigue, nausea and vomiting, and diarrhea; no clinically relevant differences in change from baseline of EORTC QLQ-C30 global health status/QoL score deterioration were observed in patients with these symptoms (data not shown). Although symptom scores were generally higher in the ribociclib plus letrozole arm during treatment and at EOT, the mean changes from baseline were less than the MID. Similar results were observed for additional EORTC QLQ-C30 questionnaire domains, including physical, emotional, cognitive, and social functioning and for breast cancer-specific EORTC QLQ-BR23 questionnaire domains, including future perspective, side effects, and upset by hair loss (Table 3).

**Table 3:** Future perspective, side effects, and upset by hair loss scores of EORTC QLQ-BR23—mean score by treatment and visit

EORTC QLQ-BR23 mean score	Future perspective		Side effects		Upset by hair loss	
	Ribociclib + letrozole *n *= 334	Placebo + letrozole *n *= 334	Ribociclib + letrozole *n *= 334	Placebo + letrozole *n *= 334	Ribociclib + letrozole *n *= 334	Placebo + letrozole *n *= 334
Baseline	41.2	42.2	14.6	15.2	15.4	19.2
Cycle 3 Day 1	49.2	51.7	21.3	17.7	27.7	30.2
Cycle 5 Day 1	54.0	55.4	20.8	17.8	34.1	29.6
Cycle 7 Day 1	53.6	57.1	20.7	17.5	37.5	33.3
Cycle 9 Day 1	56.2	59.7	21.2	17.2	39.5	35.6
Cycle 11 Day 1	54.2	58.6	21.4	17.8	42.0	33.3
Cycle 13 Day 1	58.4	59.7	21.6	18.2	36.0	34.5
Cycle 15 Day 1	58.4	64.0	21.7	16.2	39.2	30.2
Cycle 17 Day 1	58.3	64.0	20.8	17.6	36.8	35.4
Cycle 19 Day 1	58.3	63.5	21.1	16.9	34.4	27.8
Cycle 22 Day 1	63.5	62.7	20.7	17.1	34.5	30.6
Cycle 25 Day 1	57.8	64.2	21.3	17.0	30.7	26.7
EOT	44.1	46.7	24.1	19.7	37.9	30.6

*EORTC QLQ*-*BR23* European Organisation for Research and Treatment of Cancer breast cancer-specific questionnaire, *EOT* end of treatment

Only time points with data available for at least 35 patients in each treatment arm are included

For future perspective, a score of 0 = worst and a score of 100 = best. For side effects and upset by hair loss, a score of 0 = best and a score of 100 = worst. A 5–10-point improvement from baseline in EORTC score was defined as clinically meaningful

**Fig. 4:**
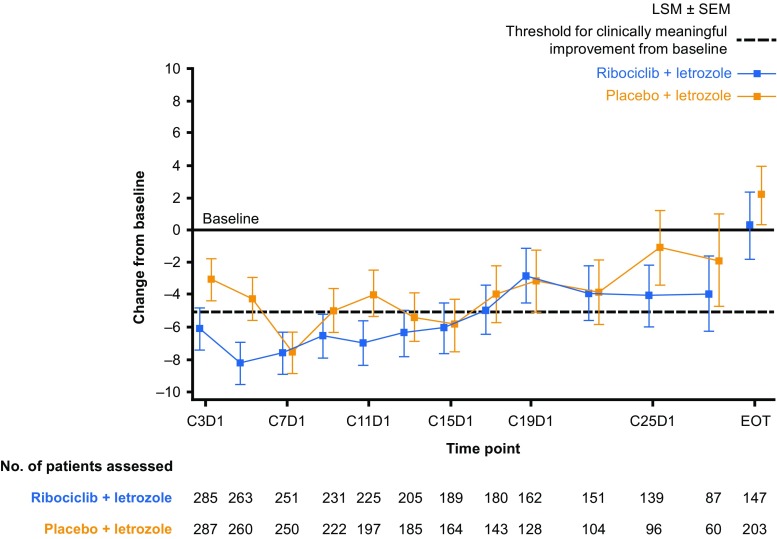
Change from baseline in EORTC QLQ-C30 pain scores by treatment arm. *C* Cycle, *D* Day, *EORTC QLQ*-*C30* European Organisation for Research and Treatment of Cancer core quality-of-life questionnaire, *EOT* end of treatment, *LSM* least squares mean, *SEM* standard error of the mean. Changes from baseline in patient-reported EORTC QLQ-C30 pain scores were determined using a linear mixed-effect model. Negative changes from baseline indicate a reduction in pain. A > 5-point change from baseline in pain score was defined as clinically meaningful. Data cut-off: January 4, 2017

**Fig. 5:**
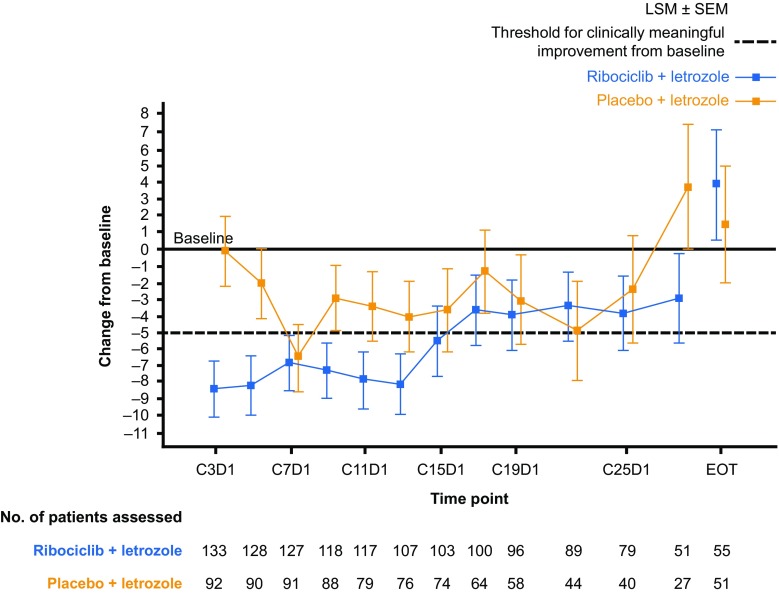
Change from baseline in EORTC QLQ-C30 pain scores in patients with a best overall response of CR or PR. *C* Cycle, *CR* complete response, *D* Day, *EORTC QLQ*-*C30* European Organisation for Research and Treatment of Cancer core quality-of-life questionnaire, *EOT* end of treatment, *LSM* least squares mean, *PR* partial response, *SEM* standard error of the mean. Changes from baseline in patient-reported EORTC QLQ-C30 pain scores were determined using a linear mixed-effect model. Negative changes from baseline indicate a reduction in pain. A > 5-point change from baseline in pain score was defined as clinically meaningful. Data cut-off: January 2, 2017

As reported previously, a clinically meaningful (> 5 points) reduction from baseline in EORTC QLQ-C30 pain score was observed as early as Week 8 in the ribociclib arm [14]. This clinically meaningful reduction in pain score was maintained up to Cycle 15 in the ribociclib plus letrozole arm (Fig. 4). Improvements of > 5 points from baseline in pain score were only observed in the placebo plus letrozole arm at Cycles 7 and 15; during all other cycles, the improvement was ≤ 5 points (Fig. 4). An improvement in pain score was also observed in the ribociclib plus letrozole arm for specific patient subgroups, including those with bone-only metastases, visceral disease (data not shown), and patients with a best overall response of CR or PR (Fig. 5).

**Fig. 6:**
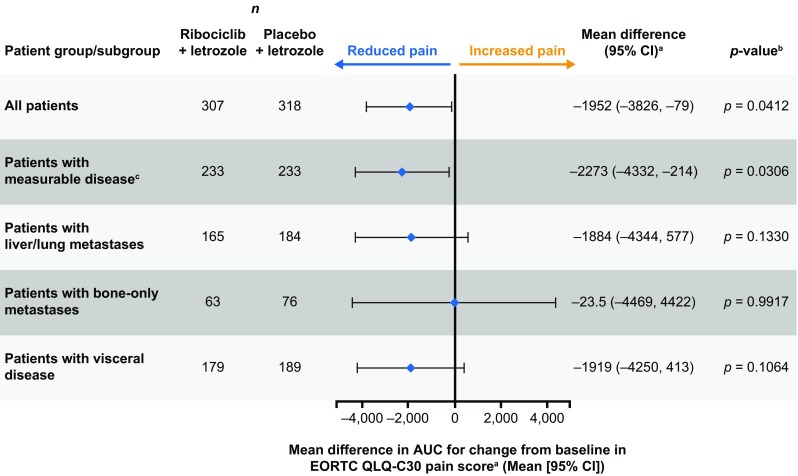
Exploratory AUC analysis for the mean difference in change from baseline in EORTC QLQ-C30 pain scores between treatment arms. *AUC* area-under-the-curve, *CI* confidence interval, *EORTC QLQ*-*C30* European Organisation for Research and Treatment of Cancer core quality-of-life questionnaire. AUC analysis for change from baseline in mean EORTC QLQ-C30 pain scores was performed for the indicated subgroups in each treatment arm. Larger negative values indicate a greater reduction in pain. ^a^Compared between treatment arms using a paired *t*-test. ^b^*p*-values reported are nominal. No multiplicity adjustments were made, and therefore, statistical interpretation should be made with caution. ^c^Measurable disease data were based on a data cut-off date of January 2, 2017. All other subgroup data were based on a data cut-off date of January 4, 2017

As conventional longitudinal analysis of PRO endpoints may not always capture the totality of the benefit throughout the treatment period, an exploratory AUC analysis for change from baseline in EORTC QLQ-C30 pain score was performed to characterize changes in pain during treatment. A reduced numeric score versus baseline represented less pain severity, whereas an increased pain score indicated greater pain severity [15]. According to the exploratory AUC analysis, a statistically significant reduction in the mean AUC-pain was observed in all patients (mean difference: −1952; 95% CI −3826, −79; *p* = 0.0412) and subgroups of patients with measurable disease at baseline (mean difference: −2273; 95% CI −4332, −214; *p* = 0.0306) in the ribociclib plus letrozole arm compared with the placebo plus letrozole arm, confirming the improvement from baseline in EORTC QLQ-C30 pain score and indicating reduced pain severity (Fig. 6).

## Discussion

Several studies have assessed the QoL of patients diagnosed with breast cancer, yet few have investigated QoL in the setting of recurrent/metastatic disease [16]. Evaluation of the impact of treatment modalities on QoL in patients with advanced disease is also limited. This study presented detailed PRO analyses for ribociclib plus letrozole in the first-line treatment of HR+, HER2− advanced breast cancer.

The association between HRQoL and PFS in oncology clinical trials has not been well characterized [17]. However, therapeutic benefit may be defined as improved efficacy in the absence of a decline in HRQoL [17]. Patient QoL is impacted by both the efficacy and tolerability profile of a therapeutic agent, and it is well known that treatment-related toxicities can adversely affect the QoL of patients with advanced breast cancer [7]. Conventional therapies such as chemotherapy can cause a number of serious adverse events, and have been shown to have a significant negative impact on QoL [18]. However, with recent advances in the treatment of metastatic/recurrent breast cancer, preserving patient QoL has become more manageable due to the availability of more tolerable agents, such as hormone therapy and CDK4/6 inhibitors [13, 19]. In addition to significantly improved efficacy with ribociclib plus letrozole versus placebo plus letrozole [4, 14], the current MONALEESA-2 analysis further demonstrates that ribociclib plus letrozole does not compromise patient QoL. HRQoL was maintained throughout the study treatment period in patients receiving ribociclib plus letrozole, but rapidly declined in both treatment arms at EOT, suggesting that HRQoL worsened in line with disease progression. In addition, a significantly greater delay in TTD in HRQoL was observed in patients without, versus with, a PFS event, suggesting that a delay in progression may help delay deterioration in HRQoL. One possible limitation of our study is the limited PRO measurement postprogression, which could have provided further insights on the impact of disease progression on HRQoL. In addition, considering the longer PFS in the ribociclib plus letrozole arm, the follow-up duration for PROs for these patients was likely to be longer versus the placebo plus letrozole arm. Despite the potential difference in follow-up, evaluation of PROs postprogression may reveal more pronounced HRQoL differences between arms and in patients with or without progression events.

In addition to maintaining overall QoL, ribociclib plus letrozole was associated with a clinically meaningful reduction in pain in the overall population, which was observed as early as Week 8 and maintained for at least 15 cycles. Significant improvements in pain score were also observed in all patients and subgroups of patients with measurable disease at baseline in the ribociclib plus letrozole arm following an exploratory AUC analysis. In a cross-sectional study involving 1072 patients with breast cancer, maintaining QoL and controlling pain were among the top 10 most important issues [20]. In addition, increasing pain severity has been associated with significant worsening of QoL in patients with advanced cancer [21]. Current guidelines suggest that assessment and management of pain is of critical importance in patients with cancer but is not adequately treated, despite recommendations that effective pain management be included as part of the treatment plan [9, 22]. Given that pain adversely impacts QoL, reducing or delaying pain symptoms could be expected to improve HRQoL. In light of this, the early improvement in pain score observed with ribociclib treatment introduces a new consideration for treatment selection in this patient population.

In conclusion, our findings demonstrated that overall HRQoL in the MONALEESA-2 study was consistently maintained from baseline in postmenopausal women with HR+, HER2− advanced breast cancer receiving ribociclib in combination with letrozole compared with placebo plus letrozole. In addition, combined ribociclib plus letrozole was associated with early and clinically meaningful improvements in pain severity compared with placebo plus letrozole. Together with the demonstrated clinical efficacy and tolerability, these PRO results provide further evidence for the benefit of ribociclib plus letrozole in this patient population.
